# The Study of Anti-/Pro-Oxidant, Lipophilic, Microbial and Spectroscopic Properties of New Alkali Metal Salts of 5-*O*-Caffeoylquinic Acid

**DOI:** 10.3390/ijms19020463

**Published:** 2018-02-04

**Authors:** Monika Kalinowska, Ewelina Bajko, Marzena Matejczyk, Piotr Kaczyński, Bożena Łozowicka, Włodzimierz Lewandowski

**Affiliations:** 1 Department of Chemistry, Biology and Biotechnology, Bialystok University of Technology, Wiejska 45E Str., 15-351 Bialystok, Poland; m.matejczyk@pb.edu.pl (M.M.); w-lewando@wp.pl (W.L.); 2 Faculty of Forestry, Bialystok University of Technology, Pilsudskiego 1A Str., 17-200 Hajnowka, Poland; ebajko@o2.pl; 3 Institute of Plant Protection—National Research Institute, Chelmonskiego 22 Str., 15-195 Bialystok, Poland; P.Kaczynski@iorpib.poznan.pl (P.K.); bozena.lozowicka@gmail.com (B.L.)

**Keywords:** chlorogenic acid, caffeoylquinic acid, spectroscopy, antioxidant, antimicrobial, lipophilicity

## Abstract

Lithium, sodium, potassium, rubidium and caesium salts of 5-*O*-caffeoylquinic acid (chlorogenic acid, 5-CQA) were synthesized and described by FT-IR (infrared spectroscopy), FT-Raman (Raman spectroscopy), UV (UV absorption spectroscopy), ^1^H (400.15 MHz), ^13^C (100.63 MHz) NMR (nuclear magnetic resonance spectroscopy). The quantum–chemical calculations at the B3LYP/6-311++G** level were done in order to obtain the optimal structures, IR spectra, NBO (natural bond orbital) atomic charges, HOMO (highest occupied molecular orbital) and LUMO (lowest unoccupied molecular orbital) orbitals and chemical reactivity parameters for 5-CQA and Li, Na and K 5-CQAs (chlorogenates). The DPPH (α, α-diphenyl-β-picrylhydrazyl) and FRAP (ferric reducing antioxidant power) assays were used for the preliminary estimation of the antioxidant properties of alkali metal chlorogenates and chlorogenic acid. In the DPPH assay the EC_50_ parameter were equal to 7.39 μM for 5-CQA and was in the range of 4.50–5.89 μM for salts. The FRAP values for two different concentrations (5 and 2.5 μM) of the studied compounds were respectively 114.22 and 72.53 μM Fe^2+^ for 5-CQA, whereas for salts they were 106.92–141.13 and 78.93–132.00 μM Fe^2+^. The 5-CQA and its alkali metal salts possess higher antioxidant properties than commonly applied antioxidants (BHA, BHT, l-ascorbic acid). The pro-oxidant action of these compounds on trolox oxidation was studied in the range of their concentration 0.05–0.35 μM. The lipophilicity (log*k_w_*) of chlorogenates and chlorogenic acid was determined by RP-HPLC (reverse phase—high performance liquid chromatography) using five different columns (C8, PHE (phenyl), CN (cyano), C18, IAM (immobilized artificial membrane)). The compounds were screened for their in vitro antibacterial activity against *E. coli*, *Bacillus sp.*, *Staphylococcus sp.*, *Streptococcus pyogenes* and antifungal activity against *Candida sp*. The 5-CQA possessed lower antibacterial (minimal inhibitory concentration, MIC = 7.06 mM) and antifungal (MIC = 14.11 mM) properties than its alkali metal salts (MIC values: 6.46–2.63 mM and 12.91–5.27mM, respectively). The synthesized chlorogenates possessed better antioxidant, lipophilic, antimicrobial as well as lower pro-oxidant properties than the ligand alone. Moreover, a systematic change of the activity of alkali metal salts along the series Li→Cs suggests that there are correlations between the studied biological properties. The type of metal cation in the carboxylate group of chlorogenate is crucial for the activity of studied compounds.

## 1. Introduction

Chlorogenic acids are mono-, di-, tri- or tetra esters of one or more cinnamic acids and quinic acid, sometimes with an aliphatic acid replacing a cinnamic acid residue. The 5-*O*-Caffeoylquinic acid (5-CQA, common name: chlorogenic acid) is one of the major chlorogenic acids with high biological importance because of its antioxidant [1,2,3], antimicrobial [4,5], neuroprotective [6], anticancer [7,8], anti-inflammatory [9] and others properties. The main source of chlorogenic acid is coffee beans where the content of 5-CQA is 7–10% of dry weight (d.w.) in the green beans of *C. canephora* (Robusta) and 5–7.5% d.w. of green beans of *C. Arabica* (Arabica) [10]. The other rich sources of chlorogenic acids are common fruits, vegetables and herbs, among others apple (12–31 mg/100 mL of apple juice) [11], potatoes (16.36 mg/100 g of fresh weight, f.w.), tomatoes (4.22 mg/100 g f.w.) [12], tea (1599 mg/100 g d.w.) [13]. Many epidemiological studies reveal a distinct correlation between the consumption of food rich in phenolic acids and a lower risk of cancer, diabetes, cardiovascular diseases and food allergies [14]. It is assumed that the high antioxidant properties are the main cause of their biological relevance and their key role in the chemoprevention of diseases. Chlorogenic acid is an effective scavenger of the OH radical (the constant rate of reaction with the radical is k = 7.73 × 10^9^ L·mol^−1^·s^−1^). ^•^OH is one of the most reactive oxygen radicals, causing damage to the cell membrane, nucleic acids, lipid oxidation and metabolic disorders [15]. Chlorogenic acid is also effective against superoxide anion radical O_2_^−^ and peroxynitrite (ONOO^−^) [16]. In the DPPH (α, α-diphenyl-β-picrylhydrazyl) assay, 5-CQA shows better antioxidant activity than common synthetic antioxidants butylhydroxyanisol (BHA), butylhydroxytoluen (BHT) or l-ascorbic acid [17]. In vitro and ex vivo studies have proven the effectiveness of chlorogenic acid in the removal of the harmful effects of the oxidative stress in cells [18]. Chlorogenic acid (in the concentration of 10 μM) protects endothelial cells of the aorta in C57BL mice against HOCl-induced oxidative stress [19]. The antioxidative mechanism of 5-CQA involves the increased production of nitric oxide and the induction of Hmox-1. The results partially explain the relationship between the consumption of products rich in phenolic compounds (including chlorogenic acids) and the reduced risk of cardiovascular diseases. In the in vitro study, chlorogenic acid protects HaCaT keratinocytes against UVB (ultraviolet B)-mediated oxidative stress [20]. The 5-CQA decreases oxidative stress induced by the presence of FeSO_2_, BHT and H_2_O_2_ in differentiated neuronal PC12 (adrenal phaeochromocytoma) cells [21]. As with many phenolic compounds, chlorogenic acid may also act as a pro-oxidant generating reactive oxygen species, i.e., superoxide anion radicals O_2_^−^, hydrogen peroxide H_2_O_2_, peroxyl radicals ROO^•^ as well as hydroxyl radicals HO^−^ produced through the Fenton reaction during the reduction of ferric ions Fe(III) to ferrous ions Fe(II). The redox inactive metals such as Al, Zn, Cd, Mg and Ca may also enhance the pro-oxidant activity of chlorogenic acid because of their stabilizing effect on the phenoxyl radical [22,23,24]. The first stable phenoxyl radical complex—iron(III) phenolate radical—was obtained by Hockert et al., in the form of a powder which was stable for over one year under dry air in the room temperature [25]. Since that, many crystal structures of metal-phenoxyl radicals were described [26]. The uncoordinated phenoxyl radicals are generally unstable and immediately change to non-radical molecules by polymerization as well as enzymatic and non-enzymatic reduction. The stabilization of the phenoxyl radicals (e.g., due to the presence of metals) causes a prolongation of their lifetime and induces the pro-oxidant activity of phenolic compounds. The dependency between the anti- and pro-oxidant properties of phenolic compounds, as well as their lipophilicity and antimicrobial activity are widely discussed in the literature [27,28,29,30,31].

The 5-CQA shows broad antimicrobial activity against gram-negative and gram-positive bacteria and yeasts [2,32,33,34,35,36,37]. The other studies showed antiviral properties of 5-CQA against Hepatitis B virus (IC_50_ = 0.4 μg/mL [38]) and Enterovirus 71 [39]. The mechanism through the 5-CQA reveals that the antimicrobial activity is not fully understood. It is assumed that 5-CQA changes the cell wall components, its permeability and releases the cell components [36,40,41,42], but the other mechanisms are discussed as well [43].

To improve or change the physical and biological activity of parent molecules, different molecular modifications can be undertaken, e.g., the synthesis of new derivatives, metal complexes or nanoencapsulation (e.g., with β-cyclodextrin, liposomes). So far several studies describing the complexing ability of chlorogenic acid toward different metal cations in aqueous solution have been published. Chlorogenic acid forms complexes with Cu(II), Mn(II), Zn(II) and Fe(III) and the formula of these compounds is ML_n_ (where L—chlorogenic acid, *n* = 1, 2 or 3) [44]. In acidic medium Cu(II) and Fe(III) chlorogenates form MCH complexes, whereas Zn(II) appears in mixed hydroxo complexes ZnL_2_OH^5−^ and ZnL_2_(OH)_6_^−^ at pH > 10. The stability of the complexes was ordered Fe > Cu > Zn > Mn. Other studies revealed that at nearly neutral pH Cu(II), Fe(II) and Mn(II) are complexed by 5-CQA with the molar ratio 1:1 [45]. Moreover, it was established that in some cases the metal cation can be reduced by phenols. It was confirmed by EPR (electron paramagnetic resonance) experiments that the paramagnetic signal of Cu(II) decreases in the presence of phenols, probably due to the reduction of Cu(II) to Cu(I) and the oxidation of phenols. In aqueous solution, 5-CQA may form various oligomeric structures because of the possibility to bind metal cations through catechol and carboxylate moieties [46]. According to Milić et al., for aqueous solution at pH 7.5 the stoichiometry is 1:1 for Pb(II) 5-CQA and 1:1 and 1:2 for Cu(II) 5-CQA [47]. The Pb(II) complex with chlorogenic acid was studied by the DFT method and electronic absorption spectrometry. Two probable structures were identified, the chelated complex 1:1 [PbL(H_2_O)_3_]^+^ where the metal cation is coordinated through the carboxylate group and the 2:1 complex where additionally the catechol moiety takes part in the coordination. The simultaneous coordination by these two group was also proposed by Naso et al., for oxidovanadium (IV) complex of chlorogenic acid with the formula Na[VOL(H_2_O)]·4H_2_O which was obtained in the solid state [48]. This complex was described by FT-IR (infrared), EPR, UV/VIS, thermogravimetric elemental analysis and its good antioxidant (toward O_2_^−^, OH^−^ and ROO^−^) and cytotoxic activities toward SKBR3 cancer cell lines were described.

In this work the alkali metal salts of chlorogenic acid were synthesized and described. So far these compounds have not been obtained in solid state and have not been studied for their spectroscopic and biological activities. It should be mentioned that the 5-CQA complexation with sodium in water solution was studied by U*V*/*V*IS spectroscopy [49] and the crystal form of caffeine-potassium chlorogenate complex was derived from coffee beans [50]. The synthesis of alkali metal salts of chlorogenic acid is intended to obtain new compounds with increased solubility in water medium and different biological activity compared to the ligand. The spectroscopic (FT-IR, FT-Raman, UV, ^1^H (400.15 MHz), ^13^C (100.63 MHz) and 2D (COSY (correlation spectroscopy), HSQC (heteronuclear single quantum correlation), HMBC (heteronuclear multiple bond coherence) NMR) as well as theoretical studies using Gaussian program [51] (including geometry, IR, NBO (natural bond orbital) atomic charges, the highest occupied molecular orbital and lowest unoccupied molecular orbital and selected chemical reactivity parameters [52]) at the B3LYP/6-311++G** level were carried out. The antimicrobial, antioxidant (by DPPH and FRAP (ferric reducing antioxidant power) assays according to [53]), pro-oxidant (as an oxidation of trolox [54]) and lipophilic properties [55] of alkali metal chlorogenates and chlorogenic acid were determined.

## 2. Results

### 2.1. Spectroscopic Study

The FT-IR and FT-Raman spectra of alkali metal chlorogenates are shown in Figures S1 and S2. The wavenumbers and intensities of the bands from the spectra of these compounds are gathered in Table 1. The assignments were done on the basis of the theoretical spectra calculated in the B3LYP/6-311++G** level for Li, Na and K chlorogenates as well as previous assignment for chlorogenic acid [2]. In the FT-IR spectra of chlorogenates, the disappearance of the bands derived from the stretches of the carbonyl group from the carboxylic moiety ν(C=O)_COOH_ was observed. This means that the alkali metal ions interact with the ligand through the carboxylate anion of the quinic acid part. In the spectra of chlorogenates new bands derived from the vibrations of the carboxylate anion were present, i.e., ν_as_(COO^−^): 1596–1603 cm^−1^ (IR), 1598–1606 cm^−1^ (R), ν_s_(COO^−^): 1385-1395 cm^−1^ (IR), oop(COO^−^): 613–615 cm^−1^ (IR). What is important, is that the effect of metal ions was seen via the changes in the wavenumbers of the bands assigned to the quinic and caffeic acid moieties.

The electronic absorption spectra UV of chlorogenates registered in methanol and water are shown in Figures S3 and S4. In the spectra, two maxima with shoulders occurred, the locations of were very close to those in the spectra of chlorogenic acid, i.e., 215–217 and 326–327 (in methanol) as well as 216 and 222–224 nm (in water). Both bands were assigned to the π⟶π* transition within the aromatic ring and the carbon C=C chain [2]. In the acid spectra, these bands were at 218 and 329 nm (in methanol) as well as 216 and 324 nm (in water). In methanolic solution the differences between the location of the corresponding bands were slightly higher compared with the spectra recorded in solution. Despite this, in the aqueous and methanolic solution the effect of the alkali metal ions on the electronic system of the ligand can be considered negligible because of the ionic nature of alkali metal salts of chlorogenic acid.

**Figure 1:**
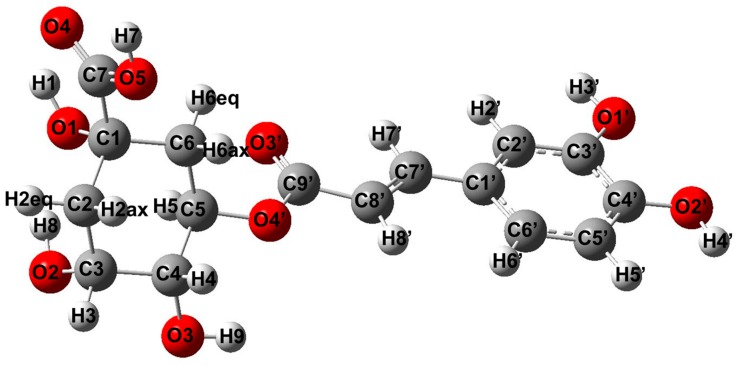
The calculated in B3LYP/6-311++G** level structure of chlorogenic acid with atom numbering.

The chemical shifts from the ^1^H and ^13^C NMR spectra of chlorogenic acid and its alkali metal salts are shown in Table 2 (atom numbering in Figure 1). The assignment was done on the basis of the previously published NMR data for chlorogenic acid and the 2D (COSY, HSQC, HMBC) NMR spectra recorded for Li chlorogenate (Figure S5). Almost all of the signals from the ^1^H NMR spectra of chlorogenates were slightly shifted up-field compared with the appropriate signals from the acid spectra. The most distinct changes concern the signals assigned to the protons from the quinic ring, i.e., H2eq, H4, H6eq and H6ax. The signals derived from the protons of the caffeic acid moiety undergo minor changes. Some of the chemical shifts decrease or increase along with the alkali metal series i.e., Li⟶Na⟶K⟶Rb⟶Cs 5-CQAs (H2eq—except Li salt, H3, H6ax and H5) according to the changes in alkali metal parameters (like electronegativity, ionic radii, ionic potential). The signals assigned to the C1–C7 carbons shifted downfield compared with the ligand spectra, which suggests a decrease in the electronic charge density around these atoms. There are no regular changes along with the alkali metal series. The values of the chemical shifts of carbons from the caffeic acid moiety are almost equal to the values of chlorogenic acid. In the alkali metal salts of chlorogenic acid the effect of metals seems to be limited to the quinic acid part of the ligand.

### 2.2. Antimicrobial Activity

Table 3 presents the MIC (minimal inhibitory concentration) values obtained for 5-CQA and its alkali metal salts against selected microorganisms. Among all tested microorganisms *E. coli* shows the highest sensitivity to the applied compounds, while *Candida sp.* is characterized by the highest degree of resistance to the tested compounds. Generally, 5-CQA and its alkali metal salts show slight antimicrobial activity against the selected microorganisms. The obtained MIC values significantly exceed the MIC values of the reference antibiotics (ampicillin and kanamycin, Table 3) for the all analyzed chemicals. Generally, alkali metal chlorogenates are characterized by better antimicrobial activity than ligands alone. Among the studied alkali metal salts, K and Rb 5-CQAs possess higher activity than the ligand alone, with the lowest MIC against *E. coli* and *Streptococcus pyogenes* for Rb 5-CQA and *Staphylococcus epidermidis* for K 5-CQA.

The antimicrobial activity of 5-CQA was previously studied against e.g., Enterobacter aerogenes [33], Klebsiella pneumoniae [2], Proteus vulgaris [2], Pseudomonas aeruginosa [2], Enterococcus faecium [2], Candida albicans [2], Saccharomyces cerevisiae with MIC ranging from 5 to 10 mg/mL. Some studies reveal higher antimicrobial activity of 5-CQA with MIC value 8–64 μg/mL against Stenotrophomonas maltophilia [34], Bacillus careus [35], Enterococcus faecalis [35], Bacillus subtilis [36], Streptococcus pneumoniae [36]. The 5-CQA was active against clinically isolated Stenotrophomonas maltophilia. The MIC value ranges from 8 to 16 μg/mL [34,37]. The main mechanism of the antimicrobial activity of phenolic compounds is directly connected with the disruption of microbial membranes, enzyme interaction, or metal ion deprivation. Moreover, the structural features of phenolics and conditions of bacteria–phenolics interactions (pH and sodium chloride concentration) can lead to physiological changes in the microorganisms and cell death [4,56,57]. This was also confirmed in the work of Li et al., who investigated the antimicrobial effect of CGA chlorogenic acid against Staphylococcus aureus. They showed that chlorogenic acid inhibits the proliferation of *S. aureus* partially by affecting the cell membrane, which was evidenced by the damage of the membrane structure, the release of cytoplasmic constituents, and changes in the pH and membrane potential [58]. Wang et al. showed that 5-CQA is active toward multi-drug resistant Staphylococcus aureus (IC_50_ = 33.86 ± 5.55 μg/mL) via inhibition of the activity of sortase A [43]. The presence of the alkali metal cations as well as chloroganate ion may affect the manner of interaction with the microbial membrane compared to undissociated ligand.

### 2.3. Antioxidant (DPPH and FRAP Assays) and Pro-Oxidant (Trolox Assay) Properties

**Figure 2:**
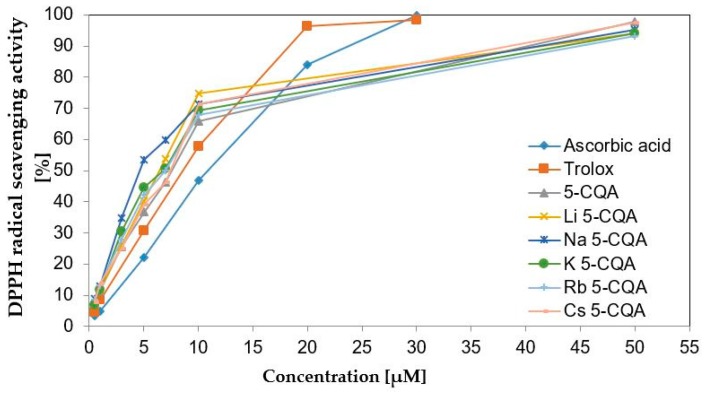
DPPH (α, α-diphenyl-β-picrylhydrazyl) radical scavenging activity (%) of chlorogenic acid, chlorogenates, trolox and ascorbic acid.

The antioxidant properties of methanolic solutions of chlorogenic acid and alkali metal chlorogenates was measured by DPPH and FRAP assays [53]. The obtained results are presented in Table 4 and Figure 2. Alkali metal salts of chlorogenic acid show slightly better antioxidant activity than the ligand alone. In the DPPH assay the EC_50_ parameter equals 7.39 μM for 5-CQA and in the range of 4.50–5.89 μM for salts. Moreover, in the DPPH assay, 5-CQA and its alkali metal salts possess higher antioxidant properties than commonly known antioxidants: Trolox (EC_50_ = 8.25 μM), l-ascorbic acid (EC_50_ = 10.87 μM) and BHT (EC_50_ = 52.80 μM). Furthermore, taking into account the increase in the EC_50_ value in the series Na⟶Li⟶K⟶Rb⟶Cs 5-CQAs⟶5-CQA a decrease in the antioxidant activity along with the mentioned series can be concluded. The antioxidant activity of the studied compounds measured in the FRAP assay increases with the increase in their concentration. The FRAP values for two different concentrations (5 and 2.5 μM) of studied compounds are respectively 114.22 and 72.53 μM Fe^2+^ for 5-CQA, whereas for salts these are 106.92–141.13 and 78.93–132.00 μM Fe^2+^. In the series of Na⟶K⟶Rb⟶Cs 5-CQAs⟶5-CQA a decrease in the ferric reducing antioxidant activity occurs. Lithium salt does not follow this rule, its antioxidant properties measured in FRAP assay are lower even than the activity of chlorogenic acid. In the FRAP assay the measurements are done for almost aqueous solutions (400 μL of methanolic sample plus 3000 μL of aqueous FRAP solution), whereas in the case of DPPH assay for the methanolic solutions. The EC_50_ value obtained in the DPPH assay for lithium salt is slightly lower than that for sodium salt, whereas the FRAP values clearly show that in the case of lithium chlorogenate some additional interactions in the aqueous solution may occur that affect the final results and caused the lithium 5-CQA to be a weaker antioxidant than the rest of the tested compounds in the FRAP assay. Because the FRAP test was conducted in aqueous media, the hydration of lithium ion may be important factor which should be taken into consideration. The lithium cation is smaller than the other alkali metal ions and the interactions between water molecules and lithium ions seems to be different than that between the water molecules and other alkali metal cations. There are data in the literature that shows that the hydration of the lithium cation from the salt molecule affects its properties and causes that lithium to not follow the period low, e.g., in the studies of hydrophobicity of alkali metal salts of warfarin and the taste response to these salts in mice [59]. According to Mähler and Persson, who studied the hydration of alkali metal ions in aqueous solution, the lithium cation may form a second hydration sphere in contrast to sodium ion and probably other heavier alkali metal cations [60].

The obtained results indicate that the studied compounds reveal antioxidant activity in both the DPPH and FRAP tests. This means that 5-CQA and its alkali metal salts are active antioxidants in the direct reaction with DPPH radical as well as having ferric reducing antioxidant power. The products of the oxidation of chlorogenic acid and chlorogenates are semi-quinones or quinones [22,23,24]. The antioxidants may react with DPPH radical via different mechanisms: (a) HAT (hydrogen atom transfer); (b) PCET (proton-coupled electron transfer); (c) SPLET (sequential proton-loss electron transfer) and ET-PT (electron-transfer proton loss) [29,61], but in polar solvents (water, methanol, ethanol) the HAT mechanism is marginal [62]. Whereas the FRAP assay is classified as totally SET (single electron transfer) reaction where one electron is transferred from the antioxidant to reduce metal cations. In the SET mechanism, two factors, the ionization potential and deprotonation, are the most significant in the evaluation of the antioxidant activity of tested compounds [63]. Taking into account the mechanism of the reactions that occur during the DPPH and FRAP assays it can be stated that chlorogenic acid and its alkali metal salts show the antioxidant activity mainly through electron transfer and the ionization potential could be one of the most important parameters defining the antioxidant potential of these compounds. The lower the ionization potential, the easier the electron abstraction. Because of the ionic character of alkali metal salts they exist predominantly in the solution as chlorogenate anion and alkali metal cations. This suggests that the ionic form of the chlorogenic acid possesses slightly higher antioxidant properties than the protonated form because of the easier electron abstraction.

**Figure 3:**
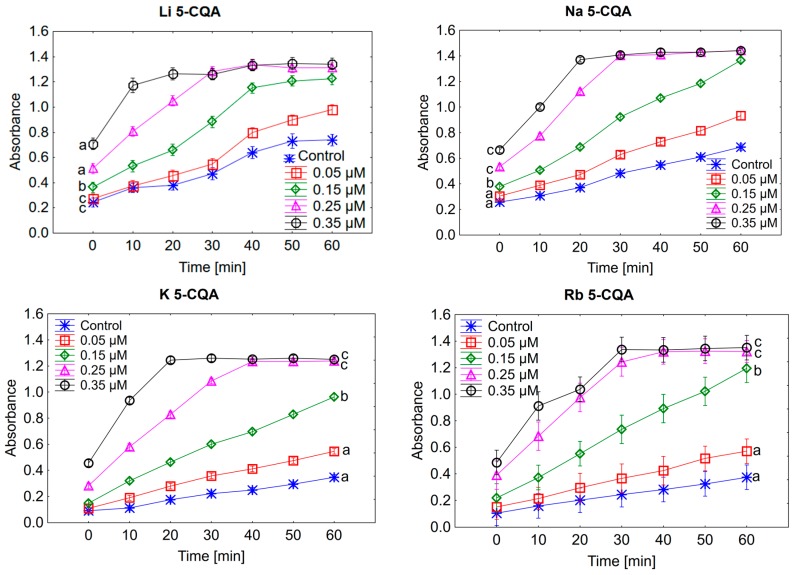

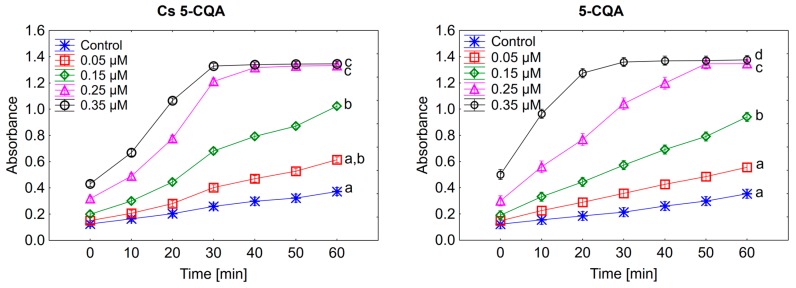
The effect of different concentrations (0.05–0.35 μM) of chlorogenic acid and its alkali metal salts on the oxidation of trolox.

**Figure 4:**
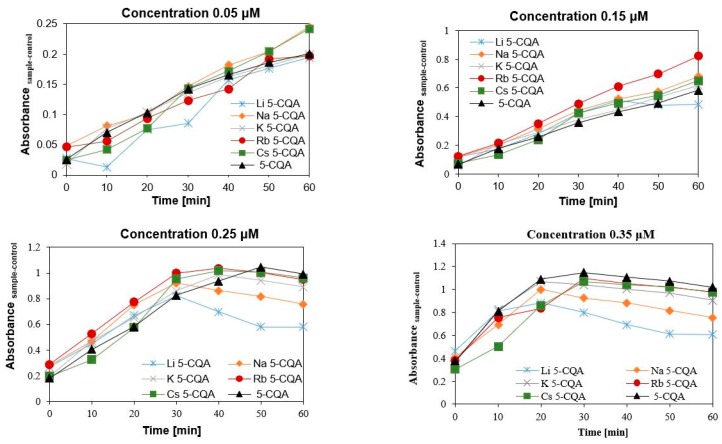
Comparison of the effect of the studied compounds at the concentration range 0.05–0.35 μM on the oxidation of trolox.

**Figure 5:**
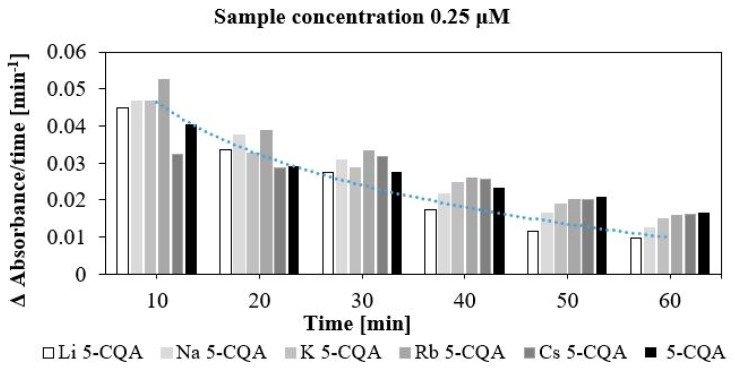

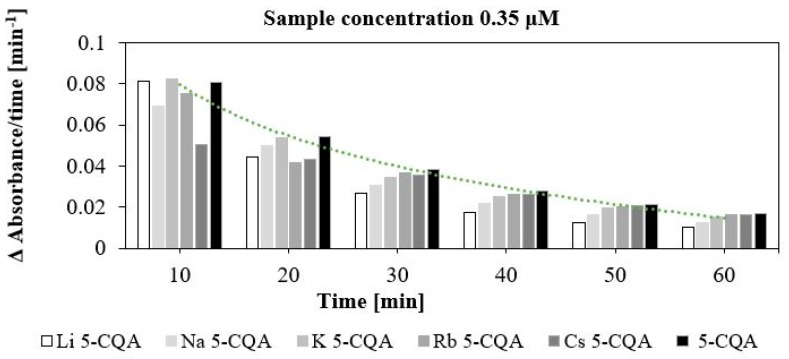
Comparison of the effect of the studied compounds at the concentration 0.25 and 0.35 μM on the oxidation rate of trolox.

Chlorogenic acid and chlorogenates were also tested for their pro-oxidative effect on trolox oxidation. The radicals of chlorogenic acid and chlorogenates are produced in their reaction with H_2_O_2_ catalysed by horse radish peroxide. Then the phenoxyl radicals react with trolox which undergoes oxidation to trolox radicals and then trolox quinones. Whereas the phenoxyl radical are transformed to phenolic compounds. The maximum absorption for trolox quinone is 272 nm. This is a general procedure to study the pro-oxidant activity of phenolic compounds [54]. In Figure 3 the effect of 5-CQA and its alkali metal salts with different concentrations of the oxidation rate of trolox is shown. The pro-oxidant effect depends on the type of compound and its concentration. With the increase in the concentration the pro-oxidant capacity of compounds increase. For the two selected concentrations, i.e., 0.05 and 0.15 μM, the oxidation of trolox did not finish after 60 min of measurement. Whereas for the last two concentrations, i.e., 0.25 and 0.35 μM, the oxidation of trolox finished after 20–50 min depending on the type of compound. In the case of 0.25 μM the reaction came to an end after 30 min. for Li and Na 5-CQAs, 40 min. for K and Rb 5-CQAs and 50 min. for chlorogenic acid. The rate of trolox oxidatation increased with another increase in concentration, i.e., in the case of 0.35 μM solution the reaction finished after 20 min for Li, Na and K 5-CQAs, and 30 min. for Rb and Cs 5-CQAs as well as 5-CQA. This can be seen very well in Figure 3, where the effect of different concentrations (0.05–0.35 μM) of chlorogenic acid and its alkali metal salts on the oxidation of trolox was depicted. Even at 60 min of measurement of the reaction course the absorbance still increases when the tested compounds are at the 0.05 and 0.15 μM (Figure 4). The maximum pro-oxidant activity is reached at a higher concentration (in our experiment: 0.25 and 0.35 μM, Figure 5) after a maximum 50 min of measurement. Then, for both the higher concentrations (0.25 and 0.35 μM) a clear trend occurs: the pro-oxidant activity of the studied compounds increases in the series of Li⟶Na⟶K⟶Rb⟶Cs 5-CQAs⟶5-CQA (Figure 5). Besides this, the presence of the catechol moiety, the type of metal in the carboxylate group of chlorogenate is crucial for the pro-oxidative properties of tested compounds. The pro-/antioxidative activity of phenolic compounds or their alkali metal salts is sometimes correlated with the increase in their lipophilicity [59].

### 2.4. Lipophilicity Studies

The chromatographic lipophilicity parameters (log*k_w_*) obtained under different chromatographic conditions are shown in Table 5. The alkyl (C18 and C8) modified silica are stationary phases commonly applied for the estimation of the lipophilicity by the use of HPLC [64]. The separation mechanism is based on mainly hydrophobic (van der Waals) interactions. In the case of the phenyl modified silica phase (PHE) the π⟶π interactions are also involved. When the more polar cyano-bonded phase (CN) is used, the additional hydrogen bond formation between cyano groups and a hydrogen atom from the hydroxyl group occurs. The IAM (immobilized artificial membrane) stationary phase is used to mimic the distribution of compounds in the phospholipid membrane. Because of the similarity of the interactions in the IAM columns to those present in the biological membrane, the lipophilicity parameters determined by the use of IAM are useful for the estimation of ADME (absorption, distribution, metabolism and excretion) properties of novel compounds [65]. Lipophilicity determined for GAPAergic compounds (compounds that pertain to or affect the neurotransmitter gamma-aminobutyric acid) on IAM column was closed to the logP estimated for phospholipid liposomes [66]. Moreover, closely related correlation between the lipophilicity determined by the use of the IAM column and the bacteriostatic and fungistatic activity of some 2,-dihydroxythiobenzanilidine was shown [67]. Taking into account data for chlorogenic acid from Table 6, the determined lipophilicity parameters (log*k_w_*) significantly differ from each other. The closest to the experimental value of logP (0.6) and theoretical logP (0.69) is the log*k_w_* determined by the use of IAM phase (log*k_w_* = 0.74). The log*k_w_* estimated by the use of other columns are higher and could be ordered as follows: C8 (1.11) < PHE (phenyl) (1.45) < CN (2.09) < C18 (3.88). The wide range of obtained log*k_w_* values is caused by the polar and hydrophilic properties of chlorogenic acid and therefore different interactions with particular column phases. In the case of alkali metal salts of chlorogenic acid, their log*k_w_* values increase in the series Li⟶Na⟶K⟶Rb⟶Cs 5-CQAs regardless of the type of column. This suggests an increase in the lipophilicity of these salts in the same order. A similar increase in the lipophilicity of alkali metal salts of warfarin (except for lithium salt) was shown in [59]. The lipophilicity parameters obtained for the CN and PHE phases (Table 5) are closely correlated to each other (the square of the correlation coefficient for the linear relationship is R^2^ = 0.9742). The high value of R^2^ was also obtained for the linear correlation between log*k_w_* determined on the C18 and IAM column, but taking into account the log*k_w_* values obtained for chlorogenic acid by the use of the C18 and IAM phases the final results are opposite (Table S1). The log*k_w_* value determined for acid in the C18 column is higher than the log*k_w_* obtained for salts, on the other hand the log*k_w_* determined for acid on IAM is lower than the values obtained for salts. The high value of log*k_w_* obtained for 5-CQA in the C18 phase indicate its high lipophilicity, whereas the low log*k_w_* determined in the IAM phase may show its hydrophilicity (which is in accordance with the properties of chlorogenic acid). Because of different forms of the occurrence of studied compounds—ionic in the case of alkali metal salts and mostly molecular in the case of acid—the obtained log*k_w_* values for chlorogenic acid and its salts should not be discussed together. On the other hand, the good correlation of the lipophilicity parameters determined in the IAM column with the experimental logP value allows one to assume that the log*k_w_* determined on this column better reflects the interactions in the phospholipid membrane.

### 2.5. DFT Studies

The geometrical parameters for chlorogenic acid and its alkali metal salts calculated for the gas phase and methanolic solution are shown in Table S2. The most visible differences between the particular compounds concern the geometry of the carboxylate anion, i.e., C7-O5 and C7-O4 bond lengths, as well as the O5-C7-O4 angle (atom numbering in Figure 5). This is not surprising, because in the series of studies of compounds a change of alkali metal cation in the carboxylate moiety occurs. Generally, the bonds between carbons and hydroxyl substituents in the quinic moiety, i.e., C1-O1, O1-H1, C3-O2, O2-H8 and C4-O3, are longer in the salt molecules compared with chlorogenic acid. This is due to the increase in the strength of the hydrogen bonds H1···O4, H8···O1 and H9···O4′ in the series 5-CQA⟶Li⟶Na⟶K 5-CQAs. This is manifested by the decrease in the lengths of the hydrogen bonds as well as a decrease in the values of the NBO atomic charges gathered on the oxygen atoms and an increase in the charges on hydrogen atoms (Tables S2 and S3). This is more explicit for the H1···O4 and H8···O1 than H9···O4′, and the hydrogen bonds H1···O4 and H8···O1 are stronger than H9···O4′. For the acid molecule, the bond H8···O1 is stronger than H1···O4. In the case of salts, the situation is the opposite, the H1···O4 becomes stronger than H8···O1, this is due to the presence of alkali metal cation in the carboxylate moiety and an increase in negative charge on the O1 atom (Δ_max_ = −0.023 e for gas phase, Δ_max_ = −0.034 e for methanol). The observed effect is mostly visible for the parameters calculated in methanolic solution than in the gas phase. 

**Figure 6:**
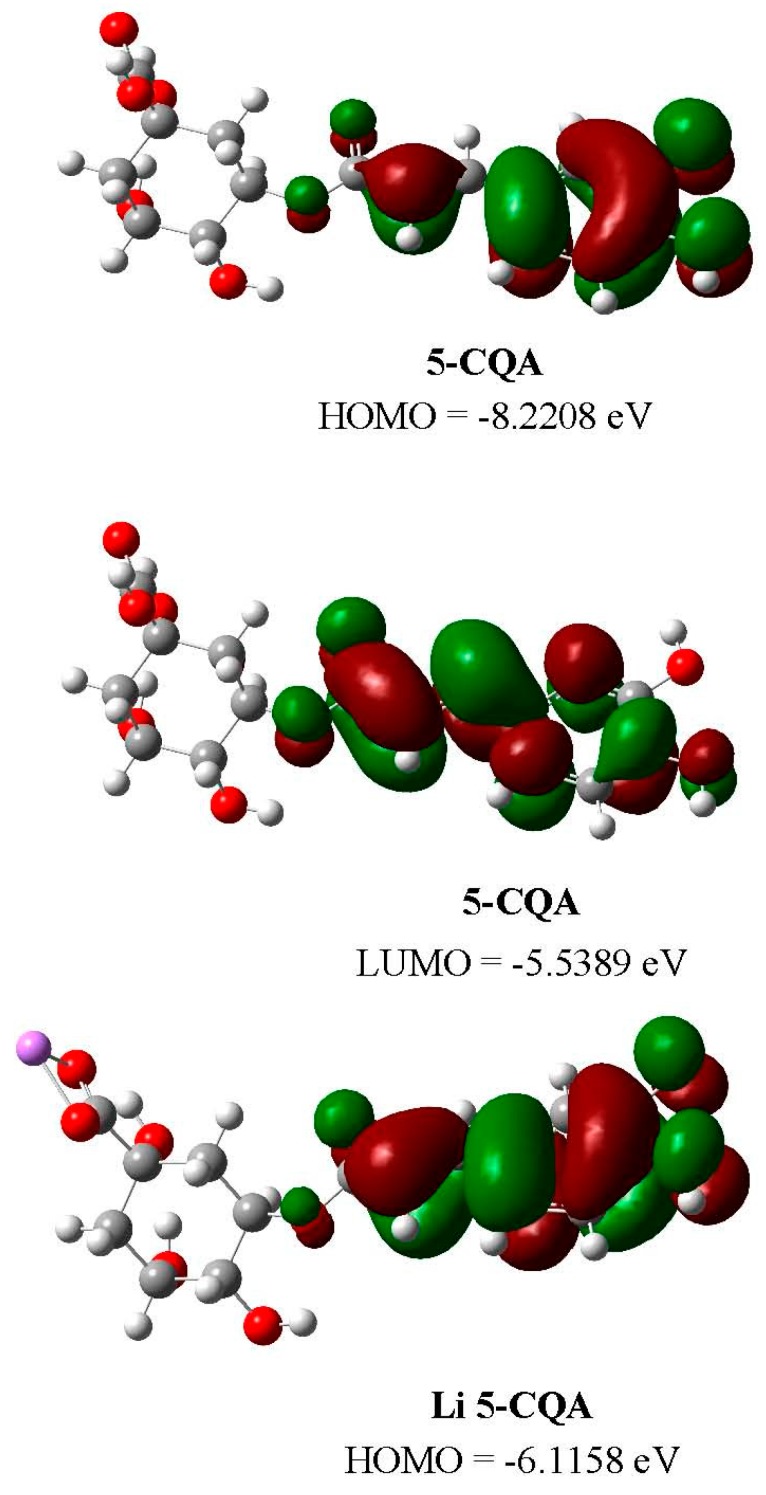

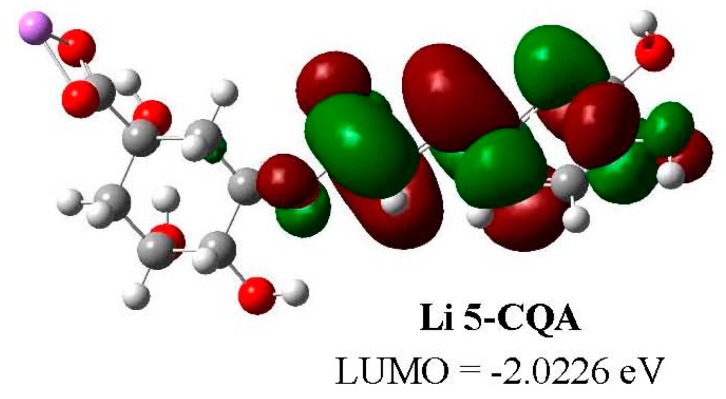
The HOMO (highest occupied molecular orbital) and LUMO (lowest unoccupied molecular orbital) orbitals calculated in gas phase 5-CQA and Li 5-CQA.

The energy parameters calculated for the studied molecules are gathered in Table 6. They are useful in the estimation of the antioxidant activity of phenolic compounds and their active site for the scavenging radical activity [37]. The HOMO and LUMO orbitals for 5-CQA and Li 5-CQA are shown in Figure 6. The HOMO energy characterizes the electron-donating ability of molecule and therefore its free radical scavenging efficiency. With the increasing values of HOMO and lowering values of ionic potential (IP), the molecules possess a rising tendency to donate electrons [37]. The calculations show that lithium, sodium and potassium salts of chlorogenic acid have a higher HOMO orbital energy and lower values of IP than chlorogenic acid. This may explain the higher antioxidant activity of chlorogenates because of their greater ability to donor electrons. Comparing the results obtained for calculations in gas and methanolic solution, the main differences concern only chlorogenic acid molecules (in gas: HOMO = −8.2208 eV, LUMO = −5.5389 eV; in methanol: HOMO = −6.1743 eV, LUMO = −2.2093 eV; Figure 6). It means that in methanolic solution 5-CQA more easily donates electrons than in the gas phase. For salt molecules there is no such tendency. The calculated values of HOMO and LUMO in gas and methanol are close each other. The HOMO energies calculated for salts in methanolic solution are slightly lower than in gas, and the ionization potential is higher. Thus the effect is opposite than for the chlorogenic acid molecule.

## 3. Discussion

The alkali metal salts of chlorogenic acid with the general formulas C_16_H_17_O_9_M·1.5H_2_O or C_16_H_17_O_9_M·2H_2_O (M—alkali metal) were synthesized. The FT-IR, FT-Raman, UV, ^1^H and ^13^C NMR methods were used to characterized the obtained compounds. The alkali metal cations interact with chlorogenic acid moiety through the carboxylate anion. The effect of metal ions on the electronic system of the ligand was seen via the changes in the number, intensities and wavenumbers of the bands assigned to the quinic and caffeic acid moieties in the FR-IR and FT-Raman spectra of chlorogenates. The experimental UV and NMR data as well as the calculation in the B3LYP/6-311++G** level for gas phase and methanolic solution show that alkali metals mainly influence the electronic charge density of the quinic part of chlorogenic acid. The presence of the hydrogen bonds in the quinic acid moiety stabilize the structure of the studied compounds. The strength of the hydrogen bonds H1···O4, H8···O1 and H9···O4′ increases in the in the series 5-CQA⟶Li⟶Na⟶K 5-CQAs. The calculations of HOMO and LUMO orbitals allows us to explain the higher antioxidant activity of chlorogenates in terms of their greater ability to donate electrons.

The antioxidant and pro-oxidant activity of chlorogenic acid depends on their concentration and the type of metal. Both DPPH and FRAP assays showed higher antioxidant activity of alkali metal salts of chlorogenic acid than chlorogenic acid alone, and the decrease in the antioxidant activity may be ordered Na⟶K⟶Rb⟶Cs 5-CQAs⟶5-CQA. Sodium salt of chlorogenic acid possesses the highest antioxidant properties. For ionic compounds, the hydration in aqueous media may affect the results obtained in the FRAP assay. The 5-CQA is a better pro-oxidant than alkali metal chlorogenates, whereas chlorogenates possess higher antimicrobial and lipophilic properties than the ligand alone. Generally, along with the series of alkali metal chlorogenates the systematic decrease in the antioxidant and an increase in the pro-oxidant, antimicrobial and lipophilic activities were reported. The results suggest that pro-oxidant, antimicrobial and lipophilic properties of chlorogenates may be closely correlated with each other. The increase in the lipophilicity of compounds means their better penetration through the cell membrane and possible better antimicrobial effects. The pro-oxidant activity of compounds may be one of the molecular mechanisms of their antimicrobial activity.

The antioxidant and pro-oxidant activities of phenolic compounds are widely studied because of their double nature and potential applications as antioxidants or pro-oxidative chemicals. The pro-oxidative effect of phenolic compounds is beneficial for their anticancer activity because the most effective anticancer agents induce apoptosis via the generation of the radical oxygen species [27,28]. Moreover, there is a clear correlation between the antimicrobial properties of plant phenolic compounds and their pro-/antioxidant activity. The results of many works carried out in the last few years have shown that there is a similar mechanism of action for different classes of bactericidal compounds [29,30,31]. This mechanism relies on the generation of hydroxyl free radicals within bacterial cells as a result of the depletion of nicotinamide adenine dinucleotide (NADH, compounds that play an essential role in cellular respiration), leaching of iron from protein iron–sulfur clusters and consequently stimulation of the Fenton reaction. Produced hydroxyl radicals cause permanent damage to protein–lipid membranes, impair cell membrane permeability, interfere with protein and DNA and consequently cause the cell death of the organisms.

The synthesized alkali metal chlorogenates revealed higher antioxidant and lower pro-oxidant properties than chlorogenic acid. Therefore, the application of chlorogenates may be more beneficial than chlorogenic acid in the case of the prevention or treatment of diseases associated with a deregulated reactive oxygen species formation. On the other hand, the increase in the pro-oxidative, antimicrobial and lipophilic activity in the series of Li→Cs chlorogenates may lead to design antimicrobial agents of natural origin with possible pro-oxidant mechanisms of action.

## 4. Materials and Methods

### 4.1. Materials Synthesis and Characterization

Chlorogenic acid, LiOH, NaOH, KOH, RbOH, CsOH, butylated hydroxytoluene (BHT), butylated hydroxyanisole (BHA), l-ascorbic acid, DPPH (2,2-diphenyl-1-picrylhydrazyl), 2,4,6-tripyridyl-*s*-triazine (TPTZ), FeCl_3_·6H_2_O, FeSO_4_, trolox, H_2_O_2_, horse radish peroxide, phosphate buffer pH = 7 were purchased from Sigma-Aldrich Co. (St. Louis, MO, USA) and used without purification. Methanol was purchased from Merck (Darmstadt, Germany).

The Li, Na, K, Rb and Cs chlorogenates were prepared by mixing the appropriate weighed mass of chlorogenic acid with an aqueous solution of alkali metal hydroxides in a stoichiometric ratio 1:1. The solutions were mixed until the acid was totally dissolved. Then, the water was evaporated with a water bath and the samples were dried at 120 °C in a dryer. The results of the elementary analysis were as follows: for lithium chlorogenate, formula C_16_H_17_O_9_Li·1.5H_2_O: %C = 49.47 (calc. %C = 49.62), %H = 5.10 (calc. %H = 5.20); for sodium chlorogenate, formula C_16_H_17_O_9_Na·1.5H_2_O %C = 47.84 (calc. %C = 47.65), %H = 4.94 (calc. %H = 5.00); for potassium chlorogenate, formula C_16_H_17_O_9_K·1.5H_2_O: %C = 45.59 (calc. %C = 45.82), %H = 4.76 (calc. %H = 4.81), for rubidium chlorogenate, formula C_16_H_17_O_9_Rb·2H_2_O: %C = 39.94 (calc. %C = 40.48), %H = 4.08 (calc. %H = 4.48); for caesium chlorogenate, formula C_16_H_17_O_9_Cs·2H_2_O %C = 35.54 (calc. %C = 36.79), %H = 3.63 (calc. %H = 4.05).

The FT-IR spectra for the solid samples were recorded in KBr matrix pellets with an Alfa Bruker spectrometer (Bremen, Germany) within the range of 400–4000 cm^−1^ with the resolution of 2 cm^−1^. The Raman spectra were registered in the range of 100–4000 cm^−1^ with a MultiRam Bruker spectrometer (Bremen, Germany) with the resolution of 1 cm^−1^. UV spectra were recorded with an Agilent Carry 5000 (Santa Clara, CA, USA) within the range of 200–400 nm. The ^1^H (400.15 MHz), ^13^C (100.63 MHz) and 2D NMR (DQF COSY: ^1^H-^1^H double quantum filtered correlation spectroscopy; HSQC; HMBC) spectra were measured on a Bruker Avance II 400 spectrometer (Bremen, Germany) in DMSO-*d*_6_ solution at 25 °C. TMS was used as an internal reference. The quantum–chemical calculations for Li, Na and K chlorogenates were done in B3LYP/6-311++G(d,p) using the GAUSSIAN 09W and GaussView software package running on a Dell PC computer (Round Rock, TX, USA) [51]. The optimized structure for chlorogenic acid was published before [2]. The geometry, IR, NBO atomic charges, the highest occupied molecular orbital (HOMO) and lowest unoccupied molecular orbital (LUMO) were calculated. The following chemical reactivity parameters were calculated: ionization potential (IP), electron affinity (EA), electronegativity (χ), chemical potential (μ), chemical hardness (η), chemical softness (σ) according the equations published in [52].

### 4.2. Evaluation of Biological Activity

Antiradical activity was conducted according to the DPPH assay described by [53]. The initial methanolic solutions of reagents were prepared at the concentration of 50 μM and 500 μM for CGA and its salt, and 60 μM for DPPH. Particular volumes of chlorogenic acid and chlorogenate solutions were added into the test tubes and diluted with methanol to obtain a series of solutions at the concentrations 10–0.1 μM (final volume 1 mL). Then, 2 mL of DPPH was added to each tube, vortexed and incubated in the darkness for 1 h at 23 °C. The final concentration of DPPH was 40 μM. The absorbance of the mixture was measured at 516 nm against methanol as the blank using Agilent Carry 5000 spectrophotometer. The control sample—2 mL of DPPH solution and 1 mL of methanol. The antiradical activity of CGA and its salts against DPPH radical was calculated according to the equation:

(1) $$\%\;I=\frac{A_{\mathrm{control}}^{516}-A_{\mathrm{sample}}^{516}}{A_{\mathrm{control}}^{516}}\times 100\text{\%}$$

where % I—% inhibition of DPPH radical, $A_{\mathrm{conrol}}^{516}$—absorbance of the control, $A_{\mathrm{sample}}^{516}$—absorbance of the sample. Then, the concentration of the tested substances was plotted against the % inhibition and the IC_50_ values were read from the scavenging curves. The IC_50_ parameter means the concentration of substance that inhibits 50% of the radical. The IC_50_ parameter was also obtained for butylated hydroxyanisol (BHA), butylated hydroxytoluene (BHT) and l-ascorbic acid and compared with those ones calculated for studied compounds.

Ferric reducing antioxidant activity was determined in FRAP assay [53]. In order to prepare the FRAP reagent the following reagents: 0.3 M acetate buffer (pH 3.6), 10 mM TPTZ (in 40 mM HCl) and 20 mM FeCl_3_·6H_2_O (in water) were mixed in a volumetric ratio 10:1:1 directly before analyses. Then, the FRAP reagent (3 mL) was mixed with the tested substance (0.4 mL; final concentrations C = 2.5 and 5.0 μM). The absorbance was measured at 594 nm against blank (3 mL of FRAP and 0.4 mL of methanol) using an Agilent Carry 5000 spectrophotometer (CA, USA). Antioxidant activity was expressed as Fe^2+^ equivalents [μM] using the calibration curve prepared over the range of 1000–20 μM concentration of FeSO_4_.

The pro-oxidant activity of the tested compounds were measured as the rate of oxidation of trolox according to the procedure described by Zeraik et al. [54]. 100 μM trolox, 50 μM H_2_O_2_, 0.01 μM horseradish peroxide in phosphate buffer (pH = 7) and tested substances with a concentration from 0.05–0.35 μM were incubated at 25 °C. The measurement were conducted at 272 nm using an Agilent Carry 5000 spectrophotometer.

The antimicrobial activity of studied compounds was tested against *Escherichia coli*, *Bacillus subtilis*, *Staphylococcus* *epidermidis*, *Streptococcus pyogenes* and antifungal activities against *Candida sp.* The microorganisms used in the experiment were obtained from the American Type Culture Collection—ATCC. Antimicrobial activity was measured as a minimal inhibitory concentration (MIC) with Müeller Hinton Agar (MHA). Serial two-fold dilutions of chemicals, ranging from 5000, 2500, 1250, 625 to 312, µg/mL were prepared. After the inoculation of microorganisms on MHA plates with certain tested chemical concentrations, the plates were incubated at 37 °C for bacteria and at 30 °C for *Candida sp.* for 24 and 48 h, respectively. Plates containing sterile water were used as controls. Minimum three repetitions were run for each assay. Ampicillin and kanamycin were used as a reference antibiotics. The MIC value was determined as the lowest concentration of chemicals, that completely inhibited bacterial and *Candida sp.* growth after 24 and 48 h of incubation. 

The lipophilicity of studied compounds was determined by RP-HPLC analysis. The Waters Alliance 2695 HPLC separation module (Milford, MA, USA) and Waters 2996 photodiode array detector (Milford, MA, USA) (λ = 254 nm) were used. The analyses were carried out on chromatographic columns: RP-18e Purospher STAR (C18), 150 × 4.0 mm, 5 μm, (Merck, Darmstadt, Germany); RP-8e Purospher STAR (C8), 150 × 4.6 mm, 5 μm, (Merck, Darmstadt, Germany); Zorbax Eclipse XDB-CN (CN), 150 × 4.6 mm, 5 μm, (Agilent Technologies, Santa Clara, CA, USA); Nucleosil Phenyl (PHE), 250 × 4.6 mm, 7 μm, (Supelco, Bellefonte, PA, USA) and Regis Rexchrom IAM.PC.DD.2 (IAM), 100 × 4.6 mm, 10 μm, (Regis Technologies Inc., Morton Grave, IL, USA). The injection volume was 10 µL and the mobile phase flow rate 1 mL min^−1^. Isocratic elution was performed with methanol-water (M-W) mixtures; C18 and C8: M-W (% *v*/*v*): 95:5, 90:10, 85:15, 80:20, 75:25; CN and PHE: M-W (% *v*/*v*): 70:30, 65:35, 60:40, 55:45, 50:50; IAM: M-W (% *v*/*v*): 75:25, 70:30, 65:35, 60:40, 55:45. The logarithm of the retention factor (log*k*) was calculated using the formula:

$$k=\frac{t_{R}-t_{0}}{t_{0}}$$

where *t_R_* is the retention time of the compound and *t*_0_ is the dead retention time determined by using a methanol solution of potassium iodide as a unretained compound. Log*k* values were extrapolated to zero methanol concentration (log*k_w_*) using the Soczewinski and Wachtmeister equation [55]:

$$\log k=S\varphi +\log k_{w}$$

where *ϕ* is the volume fraction of methanol in the mobile phase, *S* is the slope of regression plot, and log*k_w_* is the chromatographic lipophilicity.

## Figures and Tables

**Table 1:** The wavenumbers and intensities of the bands from the experimental and theoretical (in B3LYP/6-311++G** level) FT-IR (infrared) spectra of alkali metal chlorogenates and chlorogenic acid [2]; the symbols denote: ν—stretching, δ—deforming in plane and oop—out of plane bending vibrations; s—strong, m—medium, w—weak, v—very, sh—shoulders.

Li 5-CQA				Na 5-CQA				K 5-CQA				Rb 5-CQA		Cs 5-CQA		5-CQA		Assignments
IR Calc.	Int. Calc.	IR Exp.	Int. Exp.	IR Calc.	Int. Calc.	Ir Exp.	Int. Exp.	IR Calc.	Int. Calc.	IR Exp.	Int. Exp.	IR Exp.	Int. Exp.	IR Exp.	Int. Exp.	IR Exp.	Int. Exp.	
																1725	s	ν(C=O)_COOH_
1761	209.4	1687	S	1761	209.8	1694	s	1761	207.1	1692	s	1690	s	1685	s	1687	vs	ν(C=O)_ester group_
1679	204.9	1637	Sh	1680	197.7	1636	s	1681	194.9	1634	sh	1628	sh	1626	sh	1640	s	ν(C=C) + ν(CC)_ar_
1641	354.6			1642	327.5			1642	310.9							1602	m	ν(CC)_ar_ + ν(C=C)
1633	55.7			1633	55.6			1633	55.8							1613	m	ν(CC)_ar_
1581	333.4	1603	vs	1602	360.2	1598	vs	1613	326.4	1599	vs	1596	vs	1598	vs	-		ν_as_(COO^−^)
1559	134.3	1518	m	1558	134.3	1528	m	1558	135.2	1522	w	1522	m	1522	w	1530	m	ν(CC)_ar_
1462	81.9	1446	m	1460	84.8	1450	w	1465	79.8	1447	w	1447	m	1447	w	1443	m	δ(C3-H3) + δ(O2-H8)
1443	237.1	1385	sh	1423	132.3	1390	sh	1421	116.4	1395	m	1395	m	1395	m	-		ν_s_(COO^−^)
1415	4.7	1378	m	1414	8.0	1384	m	1412	28.9	1375	m	1375	m	1375	m	1382	w	δ(C-H)_quin_ + δ(O1-H1)_quin_
1304	142.8	1273	vs	1303	296.4	1281	vs	1303	326.9	1268	vs	1266	vs	1268	vs	1289	vs	ν(C4’-O2’) + δ(C-H)_ar_
1208	80.5	1184	s	1207	93.5	1178	s	1207	105.5	1181	m	1181	m	1118	m	1190	m	ν(C1’-C7’) + δ(C-H)_ar_ + ν(C3’-O1’) + δ(O2’-H4’)
1144	667.7	1119	m	1143	618.7	1119	m	1145	600.1	1119	m	1119	m	1120	m	1114	s	(C8’-H8’) + δ(O3-H9) + δ(C-H)_quin_ + δ(O1-H1)_quin_
1101	44.3	1083	w	1099	48.2	1081	m	1101	51.5	1083	w	1083	w	1083	w	1086	m	ν(C5-C6) + ν(C4-O3)
1055	16.5	1059	vw	1054	12.0	1059	w	1055	12.6					1055	vw	1057	sh	ν(C3-C3) + ν(C5-O4’) + δ(C-H)_quin_
1035	79.2	1041	w	1030	131.9	1037	w	1029	133.5	1037	w	1038	w	1037	w	1038	sh	ν(C5-O4’) + δ(C-H)_quin_
1019	28.5			1018	29.4	997	vw	1020	29.6							1000	w	oop(C7’-H7’) + oop(C8’-H8’)
973	48.3	976	w	972	49	969	w	973	50.6	974	w	976	w	976	w	977	m	ν(C1-O1) + δ(C-H)_quin_ + δ(C-H)_ar_
893	5.6	853	w	890	4.9	854	w	894	6.5	851	vw	851	vw	853	w	854	w	oop(C8’-H8’) + oop(C7’-H7’) + oop(C2’-H2’)
819	18.4	812	w	812	14.9	808	w	827	1.5	810	w	812	w	813	w	819	s	δ(C-H)_quin_ + δ(COO^−^)
790	24.3	769	w	790	23.6	772	vw	790	22.4	783	w			769	vw	768	vw	δ(C4’-C3’)
764	13.8	613	w	766	14.2	615	w	766	15.4									oop(COO^−^)
672	199.4	668	vw	670	4.1	668	wv	672	4.8	668	vw	668	vw	668	vw	670	vw	δ(C-C)_quin_
631	174.8			623	63.6	603	vw	623	63.9	603	vw	605	vw	606	vw	603	m	δ(C-C)_quin_ + oop(O5-H7)
580	18.0	571	w	581	14.8	565	vw	581	13.0	569	vw	571	vw	569	vw	564	w	δ(C-C)_ar_

**Table 2:** The chemical shifts from of ^1^H and ^13^C NMR (nuclear magnetic resonance) spectra of chlorogenic acid (5-CQA, 5-*O*-caffeoylquinic acid) and alkali metal chlorogenates (5-CQAs), δ [ppm].

Proton No.	5-CQA [2]	5-CQAs					Carbon No.	5-CQA	5-CQAs				
		Li	Na	K	Rb	Cs			Li	Na	K	Rb	Cs
H2eq	1.80	1.68	1.73	1.72	1.61	1.60	C1	73.6	75.8	75.2	75.2	75.2	75.2
H2ax	2.03	1.99	1.99	1.98	1.98	1.97	C2	37.3	39.5	39.5	39.5	39.5	39.5
H3	3.94	3.92	3.91	3.90	3.88	3.88	C3	68.2	71.4	71.4	71.5	71.6	71.6
H4	3.58	3.48	3.49	3.48	3.47	3.48	C4	70.5	71.5	71.6	71.6	71.7	71.6
H5	5.08	5.17	5.17	5.17	5.16	5.16	C5	71.0	73.2	73.3	73.4	73.5	73.5
H6eq	1.96	1.81	1.81	1.81	1.90	1.81	C6	36.4	38.0	38.1	38.2	38.2	38.2
H6ax	2.01	1.85	1.87	1.87	1.94	1.94	C7	175.0	176.5	176.7	176.5	176.2	176.0
H7	12.41						C1′	125.7	125.4	125.5	125.4	124.4	124.5
H2′	7.04	7.06	7.07	7.08	7.01	7.02	C2′	114.9	114.5	114.7	114.7	114.0	114.1
H3′	9.13						C3′	145.6	145.8	145.8	145.9	146.3	146.2
H4′	9.56						C4′	148.4	148.8	148.5	148.8	150.2	150.0
H5′	6.77	6.75	6.75	6.72	6.69	6.70	C5′	115.9	115.9	115.8	115.9	115.6	115.6
H6′	6.98	6.97	6.97	6.96	6.93	6.93	C6′	121.5	121.2	121.2	121.2	121.5	121.4
H7′	7.43	7.46	7.44	7.44	7.44	7.44	C7′	145.0	144.7	144.6	144.7	144.9	144.9
H8′	6.16	6.22	6.22	6.22	6.21	6.22	C8′	114.4	114.5	114.6	114.5	113.7	114.1

**Table 3:** MIC (minimal inhibitory concentration) values (mM) of tested compounds against selected microorganisms.

Compounds	*E. coli* (Gram−)	*Bacillus sp.* (Gram+)	*Staphylococcus Epidermidis* (Gram+)	*Streptococcus Pyogenes* (Gram+)	*Candida sp.* (Gram+)
5-CQA	7.06 (2.5 mg/mL)	˃7.06 (2.5 mg/mL)	˃7.06 (2.5 mg/mL)	˃7.06 (2.5 mg/mL)	˃14.11 (5 mg/mL)
Li 5-CQA	6.46	˃6.46	˃6.46	˃6.46	˃12.91
Na 5-CQA	6.20	˃6.20	˃6.20	˃6.20	˃12.40
K 5-CQA	5.96	5.96	2.98	˃5.96	5.96
Rb 5-CQA	2.63	5.27	5.27	2.63	5.27
Cs 5-CQA	4.79	˃4.79	˃4.79	˃4.79	˃9.58
Kanamycin (positive control)	0.21	0.21	0.21	0.21	0.41
Ampicillin (positive control)	0.43	0.57	0.57	0.57	0.57

**Table 4:** Antioxidant properties of the chlorogenic acid, chlorogenates, trolox, l-ascorbic acid and BHT expressed as the ability to scavenge 50% of the free radical DPPH· (α, α-diphenyl-β-picrylhydrazyl) (EC_50_) and FRAP (ferric reducing antioxidant power) values (at concentrations 5 and 2.5 μM).

Compound	DPPH	FRAP Values	
	EC_50_ [µM]	C_compound_ [µM]	C _Fe2+_ [µM]
5-CQA	7.39 ± 0.71	5 2.5	114.22 ± 8.09 72.53 ± 2.42
Li 5-CQA	5.28 ± 0.375	5 2.5	106.92 ± 1.28 78.93 ± 4.23
Na 5-CQA	4.50 ± 0.36	5 2.5	141.13 ± 5.78 103.08 ± 2.79
K 5-CQA	5.32 ± 0.49	5 2.5	132.00 ± 6.07 99.89 ± 7.05
Rb 5-CQA	5.40 ± 0.68	5 2.5	123.16 ± 9.56 86.70 ± 2.86
Cs 5-CQA	5.89 ± 1.57	5 2.5	117. 00 ± 6.22 80.47 ± 5.68
Trolox	8.25 ± 0.75	5	70.85 ± 3.57
l-ascorbic acid	10.87 ± 0.53	5	81.70 ± 4.23
BHT	52.80 ± 2.83	5	69.66 ± 5.54

**Table 5:** Lipophilicity parameters determined by chromatographic methods (the logarithm of the retention factor, log*k_w_*) and octanol/water partition coefficients logP obtained for chlorogenic acid and chlorogenates.

Compound	log*k_w_*						logP
	C18	C8	CN	IAM	PHE		
5-CQA	3.88	1.11	2.09	0.74	1.45	Exp.	0.6 [1]
						Calc.	−0.45 [2] 0.07 [3] 0.69 [4]
Li 5-CQA	1.84	0.21	0.84	1.24	1.23		
Na 5-CQA	2.24	1.11	1.35	1.84	1.43		
K 5-CQA	2.36	1.24	1.84	1.91	1.84		
Rb 5-CQA	3.21	1.84	2.23	2.84	2.24		
Cs 5-CQA	3.74	2.13	2.64	3.84	2.64		

**Table 6:** Calculated (in B3LYP/6-311++G** level) electronic parameters for 5-CQA and Li, Na and K 5-CQA.

Parameters	5-CQA		Li 5-CQA		Na 5-CQA		K 5-CQA	
	Gas	MeOH	Gas	MeOH	Gas	MeOH	Gas	MeOH
LUMO (hartree)	−0.2036	−0.0812	−0.0743	−0.0799	−0.0719	−0.0797	−0.0687	−0.0796
HOMO (hartree)	−0.3021	−0.2269	−0.2248	−0.2264	−0.2222	−0.2263	−0.2206	−0.2262
LUMO (eV)	−5.5389	−2.2093	−2.0226	−2.1747	−1.9554	−2.1685	−1.8697	−2.1647
HOMO (eV)	−8.2208	−6.1743	−6.1158	−6.1607	−6.0450	−6.1579	−6.0037	−6.1555
Energy gap (eV)	2.6820	3.9650	4.0931	3.9859	4.0896	3.9895	4.1340	3.9908
Ionisation potential (eV)	8.2208	6.1743	6.1158	6.1607	6.0450	6.1579	6.0037	6.1555
Electron affinity (eV)	5.5389	2.2093	2.0226	2.1747	1.9554	2.1685	1.8697	2.1647
Electronegativity (eV)	6.8799	4.1918	4.0692	4.1677	4.0002	4.1632	3.9367	4.1601
Electronic chemical potential (eV)	−6.8799	−4.1918	−4.0692	−4.1677	−4.0002	−4.1632	−3.9367	−4.1601
Chemical hardness (eV)	1.3410	1.9825	2.0466	1.9930	2.0448	1.9947	2.0670	1.9954
Chemical softness (eV)	0.3729	0.2522	0.2443	0.2509	0.2445	0.2507	0.2419	0.2506
Electrophilicity index (eV)	17.6485	4.4316	4.0454	4.3578	3.9128	4.3445	3.7488	4.3365
Total energy (a.u.)	−1297.950	−1297.985	−1304.95	−1305.029	−1459.7200	−1459.795	−1897.370	−1897.433
Dipole moment (Debay)	6.1992	8.6622	9.0114	13.4233	11.7279	15.4030	14.0710	17.1000

## Supplementary Materials

Supplementary materials can be found at http://www.mdpi.com/1422-0067/19/2/463/s1.
