# Supplementary material for: The Study of Anti-/Pro-Oxidant, Lipophilic, Microbial and Spectroscopic Properties of New Alkali Metal Salts of 5-O-Caffeoylquinic Acid
Source: Int J Mol Sci. 2018 Feb 4;19(2):463. doi: 10.3390/ijms19020463 (PMC5855685; doi:10.3390/ijms19020463)
Supplement: Supplementary file 1 [file ijms-19-00463-s001.pdf]

Supplementary Materials

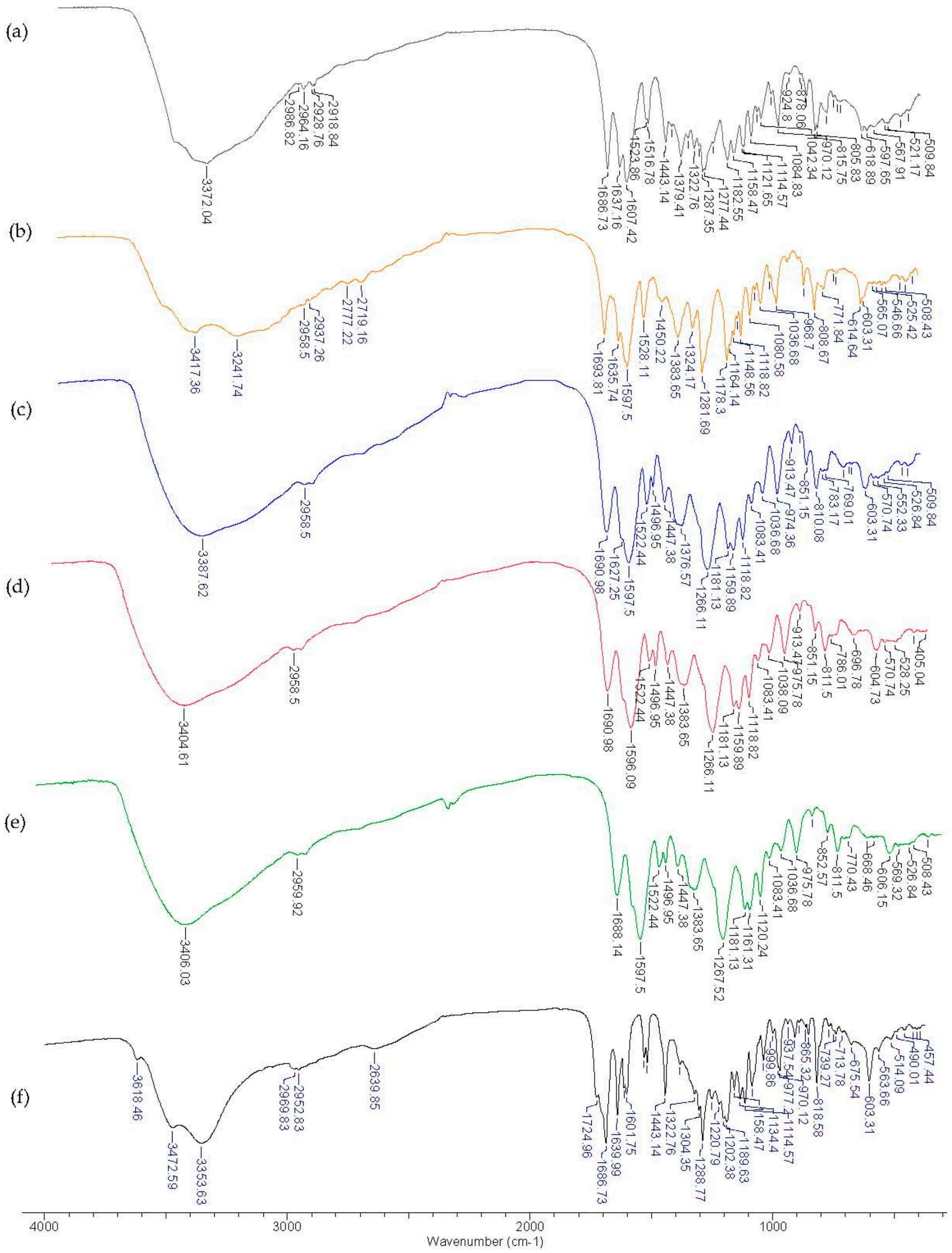

Figure S1. FT-IR spectra of (**a**) lithium; (**b**) sodium; (**c**) potassium; (**d**) rubidium; (**e**) caesium chlorogenates and (**f**) chlorogenic acid.

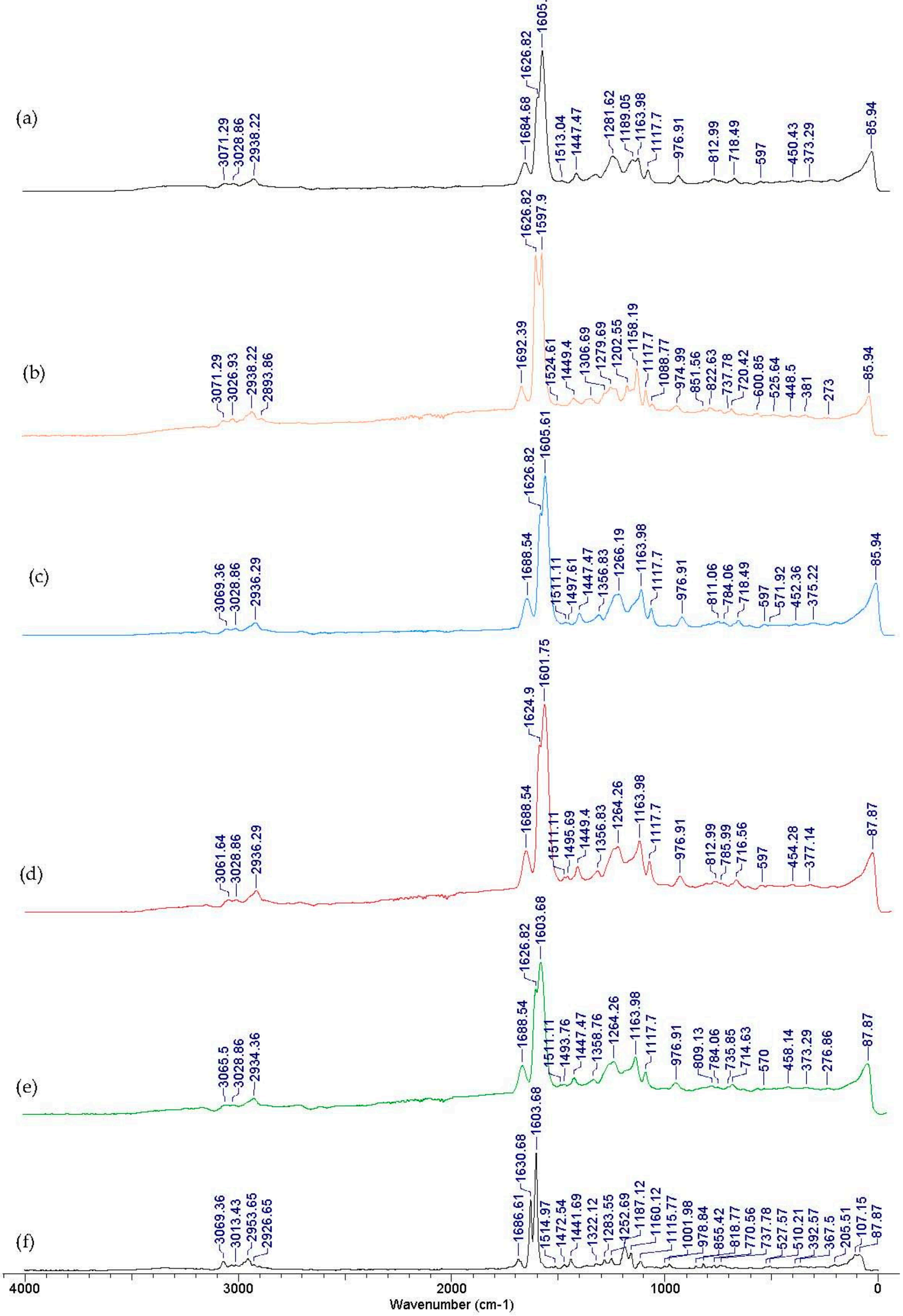

Figure S2. FT-Raman spectra of (**a**) lithium; (**b**) sodium; (**c**) potassium; (**d**) rubidium; (**e**) caesium chlorogenates and (**f**) chlorogenic acid.

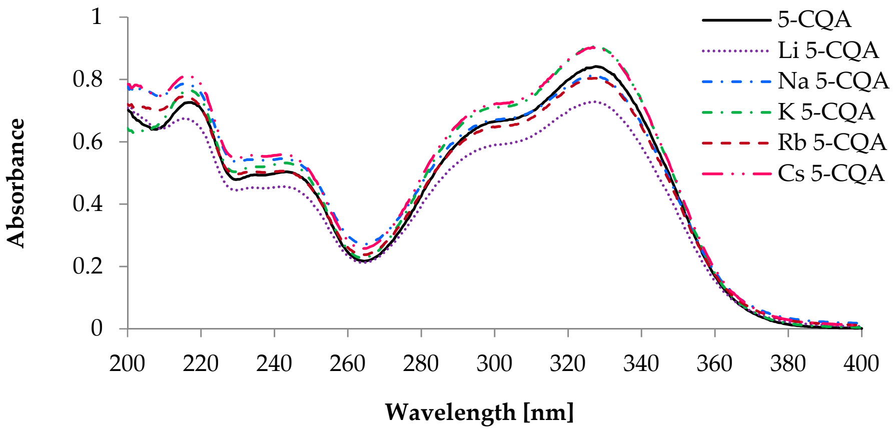

Figure S3. The UV spectra of alkali metal chlorogenates and chlorogenic acid registered in methanolic solution.

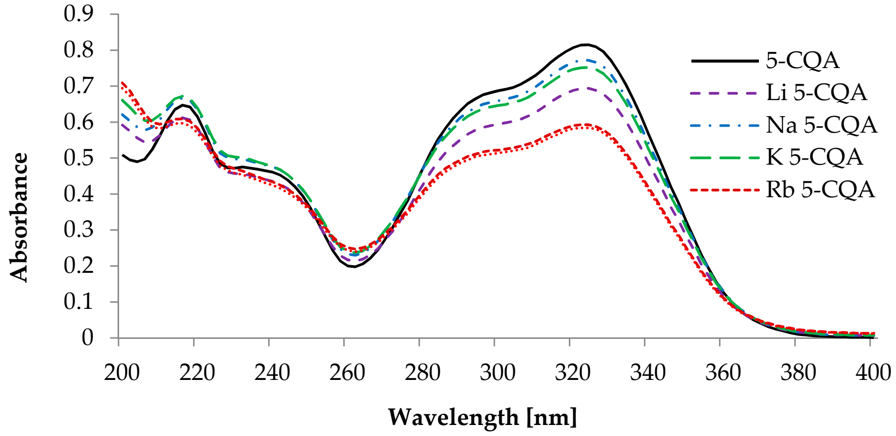

Figure S4. The UV spectra of alkali metal chlorogenates and chlorogenic acid registered in aqueous solution.

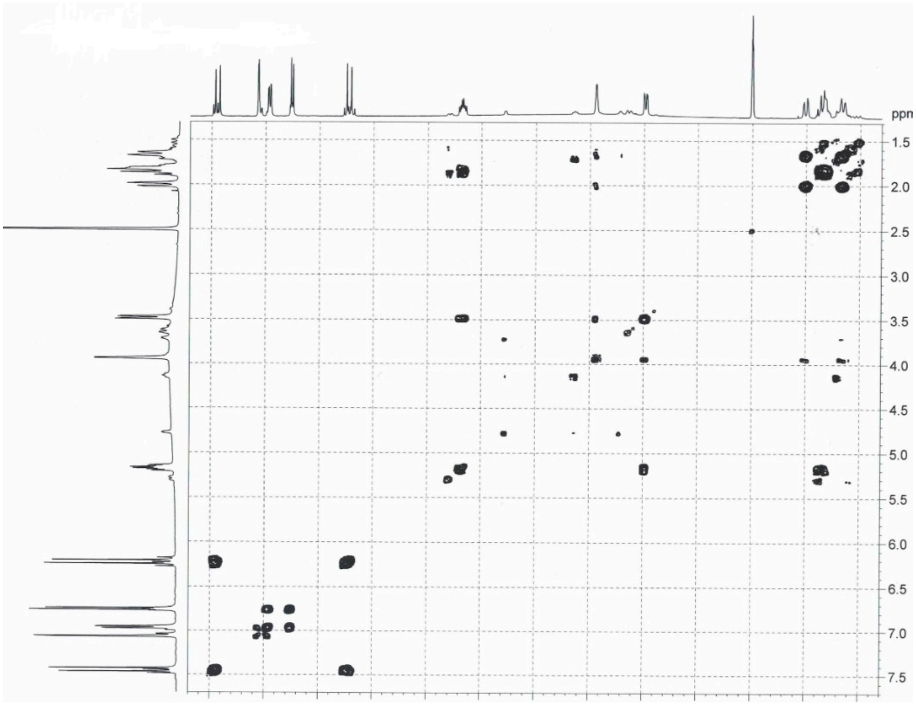

(**a**)

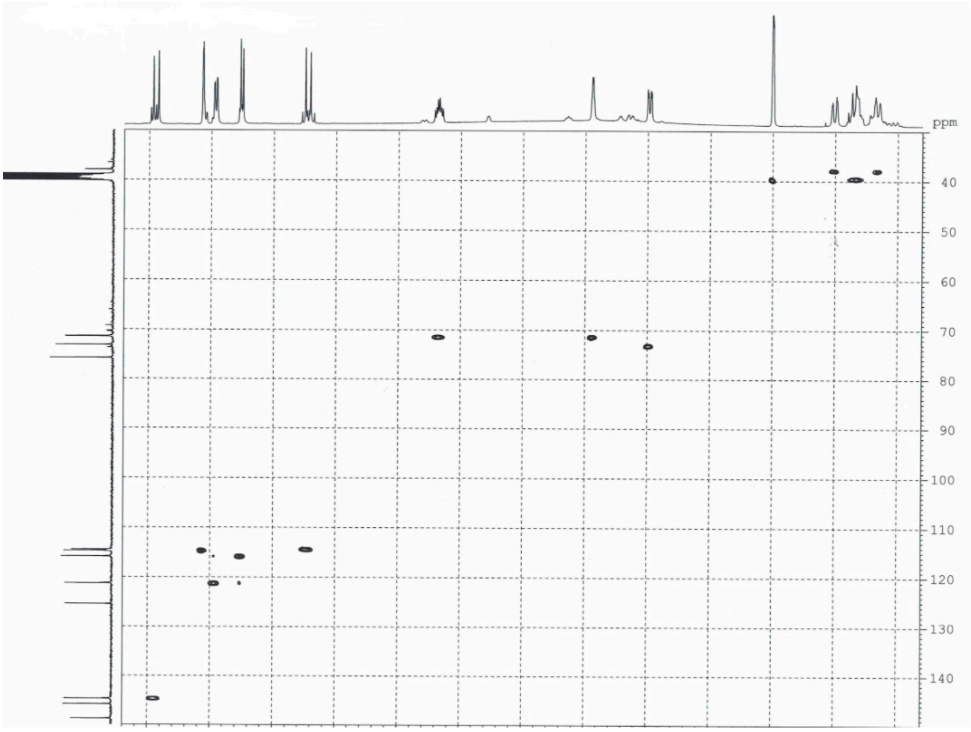

(**b**)

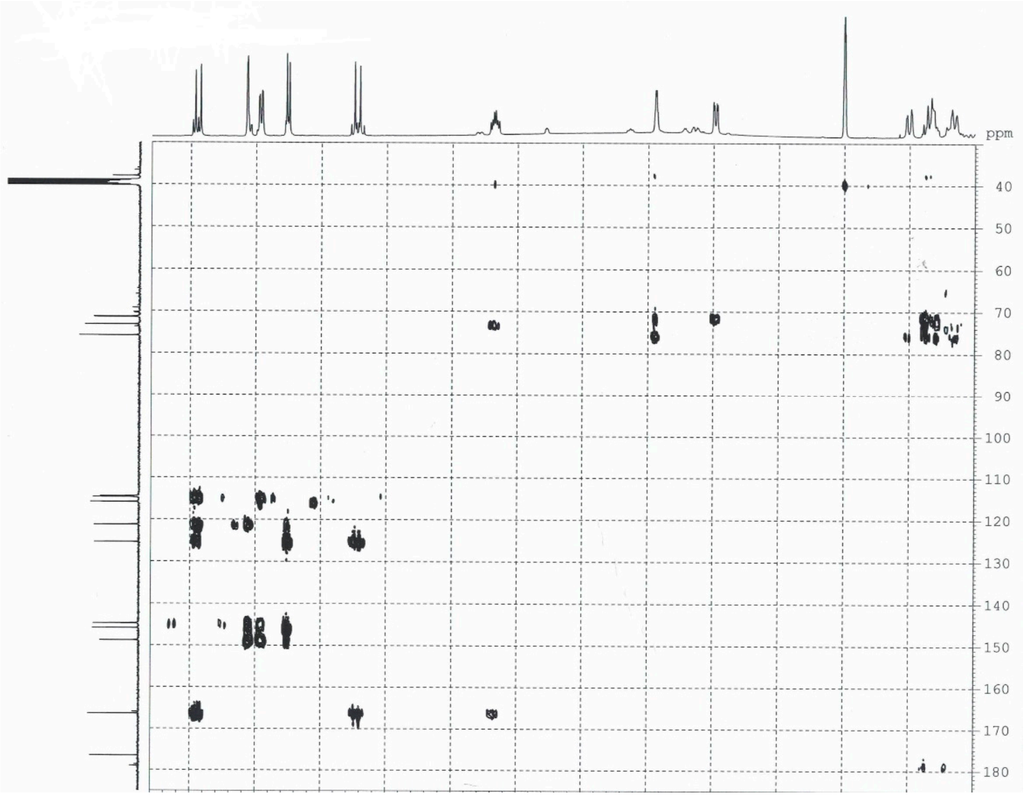

(**c**)

Figure S5. 2D (**a**) COSY; (**b**) HSQC; (**c**) HMBC NMR spectra recorded for Li chlorogenate.

Table S1. The square of the correlation coefficients for the linear relationship between logk_w determined by chromatographic method for chlorogenic acid and chlorogenates.

	C18	C8	CN	IAM	PHE
C18	-				
C8	0.8982	-			
CN	0.9184	0.9537	-		
IAM	0.9865	0.8782	0.8991	-	
PHE	0.9578	0.8930	0.9742	0.9300	-

Table S2. Selected bond distances [Å], angles [°] calculated for 5-CQA and Li, Na and K 5-CQAs in gas phase and methanol in B3LYP/6-311++G**.

Distance [Å]	5-CQA		Li 5-CQA		Na 5-CQA		K 5-CQA	
	Gas	MeOH	Gas	MeOH	Gas	MeOH	Gas	MeOH
C7-O5	1.208	1.334	1.263	1.255	1.259	1.253	1.257	1.252
C7-O4	1.344	1.211	1.274	1.266	1.270	1.266	1.270	1.266
C1-O1	1.429	1.434	1.433	1.441	1.435	1.443	1.435	1.443
O1-H1	0.969	0.971	0.971	0.975	0.973	0.978	0.974	0.979
C3-O2	1.417	1.429	1.419	1.432	1.420	1.432	1.420	1.432
O2-H8	0.967	0.970	0.968	0.971	0.969	0.972	0.969	0.972
C4-O3	1.415	1.423	1.417	1.426	1.418	1.426	1.419	1.426
O3-H9′	0.964	0.965	0.963	0.965	0.963	0.965	0.963	0.965
C9′-O4′	1.366	1.367	1.362	1.359	1.360	1.358	1.359	1.358
C9′-O3′	1.210	1.216	1.211	1.217	1.211	1.217	1.211	1.217
C8′-C7′	1.349	1.347	1.344	1.347	1.344	1.347	1.343	1.347
C3′-O1′	1.364	1.367	1.364	1.367	1.365	1.367	1.364	1.367
O1′-H3′	0.963	0.965	0.963	0.965	0.963	0.965	0.963	0.965
C4′-O2′	1.360	1.361	1.361	1.361	1.362	1.362	1.362	1.362
O2′-H4′	0.963	0.965	0.963	0.965	0.963	0.965	0.963	0.965
**Angles [°]**								
O5-C7-O4	123.43	124.26	121.70	123.84	124.19	125.56	125.03	126.27
C1-O1-H1	107.99	107.63	106.37	104.43	105.30	103.59	104.64	103.14
C3-O2-H8	108.50	107.61	108.08	107.23	107.85	107.02	107.79	107.04
C4-O3-H9	107.55	107.66	107.41	107.59	107.32	107.57	107.26	107.55
O3′-C9′-O4′	123.09	122.87	123.44	123.06	123.67	123.13	123.76	123.13
H7′-C7′-C8′-H8′	−179.76	−179.75	−179.71	−179.83	179.65	−179.89	179.80	−179.80
O1′-C3′-C4′-O2′	−0.034	−0.134	−0.054	−0.018	−0.001	−0.063	−0.016	−0.065
H3′-O1′-C3′	109.62	109.85	109.56	109.81	109.50	109.80	109.48	109.79
H4′-O2′-C4′	109.66	110.02	109.58	110.01	109.57	109.97	109.52	109.94
**Hydrogen Bonds [Å]**								
H1···O4	2.042	2.047	1.963	1.901	1.924	1.868	1.903	1.853
H8···O1	1.978	1.941	1.957	1.909	1.939	1.893	1.937	1.899
H9···O4′	2.377	2.440	2.369	2.442	2.365	2.433	2.357	2.425

Table S3. Selected NBO atomic charges [e] calculated for 5-CQA and Li, Na and K 5-CQAs in gas phase and methanol in B3LYP/6-311++G**.

Atomic Charges [e]	5-CQA		Li 5-CQA		Na 5-CQA		K 5-CQA	
	Gas	MeOH	Gas	MeOH	Gas	MeOH	Gas	MeOH
O4	−0.608	−0.631	−0.846	−0.810	−0.834	−0.800	−0.835	−0.822
O5	−0.677	−0.671	−0.818	−0.825	−0.805	−0.823	−0.806	−0.799
C7	0.865	0.816	0.771	0.770	0.770	0.771	0.773	0.773
C1	0.189	0.191	0.189	0.186	0.190	0.186	0.186	0.183
O1	−0.778	−0.787	−0.789	−0.812	−0.797	−0.818	−0.801	−0.821
H1	0.497	0.502	0.500	0.504	0.501	0.504	0.501	0.503
C3	0.119	0.115	0.119	0.116	0.119	0.116	0.119	0.116
O2	−0.740	−0.774	−0.746	−0.782	−0.750	−0.784	−0.751	−0.785
H8	0.481	0.488	0.488	0.490	0.484	0.491	0.484	0.491
C4	0.100	0.094	0.100	0.094	0.100	0.095	0.100	0.096
O3	−0.721	−0.752	−0.724	−0.754	−0.725	−0.755	−0.726	−0.755
H9	0.468	0.478	0.462	0.477	0.461	0.475	0.460	0.476
C3′	0.272	0.264	0.271	0.264	0.271	0.264	0.271	0.264
O1′	−0.657	−0.685	−0.658	−0.685	−0.658	−0.685	−0.659	−0.685
H3′	0.469	0.489	0.468	0.489	0.468	0.489	0.468	0.489
C4′	0.298	0.291	0.290	0.294	0.289	0.290	0.288	0.290
O2′	−0.649	−0.675	−0.651	−0.676	−0.652	−0.676	−0.652	0.676
H4′	0.469	0.491	0.468	0.491	0.468	0.491	0.468	0.491
